# Determining the factors impacting the quality of life among the general population in coastal communities in central Vietnam

**DOI:** 10.1038/s41598-024-57672-0

**Published:** 2024-03-24

**Authors:** Gia Thanh Nguyen, Thang Binh Tran, Duong Dinh Le, Tu Minh Nguyen, Hiep Van Nguyen, Phuong Uyen Ho, Son Van Tran, Linh Nguyen Hoang Thuy, Trung Dinh Tran, Long Thanh Phan, Thu Dang Thi Anh, Toru Watanabe

**Affiliations:** 1 Faculty of Public Health, Hue University of Medicine and Pharmacy, Hue University, 06 Ngo Quyen Street, Hue City, Thua Thien Hue Vietnam; 2 Undergraduate Training Office, Hue University of Medicine and Pharmacy, Hue University, Hue City, Vietnam; 3 Population Health Sciences Institute, Newcastle University, Newcastle upon Tyne, UK; 4 Department of Food, Life and Environmental Sciences, Yamagata University, Yamagata, 997-8555 Japan; 5 Faculty of Public Health, Da Nang University of Medical Technology and Pharmacy, Da Nang, Vietnam

**Keywords:** Quality of life, Coastal area, Flood, WHOQOL-BREF, Health policy, Public health, Quality of life

## Abstract

People living in coastal areas are frequently affected by natural disasters, such as floods and storms. This study aimed to assess the quality of life (QoL) of people living in disadvantaged coastal communes (subdivision of Vietnam) and identify their associated factors by using the World Health Organization’s quality of life instrument (WHOQOL-BREF). To achieve this, a cross-sectional descriptive study was conducted on 595 individuals aged 18 years and above living in the coastal communes in Thua Thien Hue province, Vietnam, from October 2022 to February 2023. The results showed that the mean overall QoL (mean ± SD) was 61.1 ± 10.8. Among the four domains of QoL, the physical health (57.2 ± 12.3) domain had a lower score than the psychological health (61.9 ± 13.0), social relations (63.4 ± 13.4), and environment (61.9 ± 13.3) domains. The QoL score of the domains for participants affected by flooding was significantly lower than that of those not affected, except for social relations. Multivariable logistic regression showed that subjects with not good QoL had the educational background with no formal education (Odds ratio (OR) = 2.63, 95% CI 1.19–5.83), fairly poor/poor households (OR = 2.75, 95% CI 1.48–5.12), suffered Musculoskeletal diseases (OR = 1.61, 95% CI 1.02–2.56), unsatisfaction with health status (OR = 5.27, 95% CI 2.44–11.37), family conflicts (OR = 4.51, 95%CI 2.10–9.69), and low levels of social support (OR = 2.62; 95% CI 1.14–6.02). The analysis also revealed that workers (OR = 0.17, 95% CI 0.04–0.66) had a better QoL than farmer-fisherman. QoL in disadvantaged coastal communes was low, with the lowest scores in the physical health domain. Based on the socioeconomic factors associated with not good QoL identified here, it is recommended that local authorities take more appropriate and practical measures to increase support, including measures for all aspects of physical health, psychological health, social relations, and the living environment, especially for people affected by floods.

## Introduction

Vietnam has a 3260 km coastline and is affected by a tropical monsoon environment that produces 12–14 typhoons annually^1,2^. Fifty percent of the major cities in Vietnam, accounting for 31% of the national population, are located on the coast and are vulnerable to frequent natural disasters such as storms, floods, and coastal erosion, which escalate under climate change^3^. It is crucial to consider a wide range of elements that affect the quality of life (QoL) in coastal areas because each location has distinctive socioeconomic characteristics, cultural identities, and environmental conditions^4^. Policymakers should establish initiatives to improve the well-being of coastal populations by understanding the multidimensional nature of QoL and its determinants^5^.

According to the World Health Organization, QoL refers to an individual's feelings about their life based on their goals, expectations, standards, and concerns^6^. In health and medicine, QoL has been established as an important concept and goal in both research and practice^7^. Popular criteria that can be chosen for evaluation include the level of physical and mental satisfaction with social relationships and the living environment^8^. The number of people living below the poverty line is higher in Vietnam’s coastal areas than in other regions, and national health standards are either unmet or below the national average^9^. Moreover, residents living in these areas usually have to endure various problems, such as access to hygienic water and transportation difficulties^9^. Despite the difficulties and challenges in the QoL of people living along coastlines, there is a lack of reported investigations on this topic in Vietnam, despite the country having one of the longest coastlines in Asia^10^.

Researchers have investigated floods in Vietnam and their impacts on property loss, death, illness, and other risks^11,12^. Thua Thien Hue Province, which is the site of this study, has recently experienced natural disasters with significantly increased intensity and frequency, resulting in significant socioeconomic losses and possible negative impacts on the environment and QoL of residents^13^. However, few studies have assessed the impact of floods on QoL, especially for people living in coastal areas, who are more vulnerable.

This study aimed to assess the QoL among people living in the coastal communes (subdivision of Vietnam) of a province in central Vietnam and identify their associated factors by using the World Health Organization’s quality of life instrument (WHOQOL-BREF). Understanding these factors will enable policymakers to design and implement targeted interventions that shed light on the vulnerabilities and QoL of subjects.

## Results

### General characteristics

Table 1 shows the general characteristics of the participants and their associations with the overall QoL. Female participants constituted 53.9%, while those aged 60 years and older accounted for 37.0%. Most participants had attended primary school (32.8%), followed by secondary school (31.4%). Most study participants were non-religious and married, accounting for 78.8% and 90.4% of the sample, respectively. The main profession of the research participants was farming fishermen (47.1%). In addition, research participants living in poor (6.6%) and near-poor (9.9%) households remained. And 16.1% of them were unsatisfied with their current health status. More than half (57.0%) and 19.0% of the study population was affected by storms and floods, respectively. A high level of social support was reported by 91.4% of the participants, while 7.9% experienced family conflicts. Exposure to potentially harmful substances during work was reported by 17.1% of participants.

**Table 1:** General characteristics of the research subjects (n = 595) and the association with overall quality of life.

	Number of subjects	Overall quality of life		*p*
		Not good	Good	
Gender				
Male	274 (46.1)	162 (59.1)	112 (40.9)	0.475
Female	321 (53.9)	199 (62.0)	122 (38.0)	
Age group				
18–29	59 (9.9)	29 (49.2)	30 (50.8)	**< 0.001**
30–39	90 (15.1)	42 (46.7)	48 (53.3)	
40–49	79 (13.3)	45 (57.0)	34 (43.0)	
50–59	147 (24.7)	87 (59.2)	60 (40.8)	
≥ 60	220 (37.0)	158 (71.8)	62 (28.2)	
Educational background				
No formal education	102 (17.1)	81 (79.4)	21 (20.6)	**< 0.001**
Primary school	195 (32.8)	128 (65.6)	67 (34.4)	
Secondary school	187 (31.4)	108 (57.8)	79 (42.2)	
High school and above	111 (18.7)	44 (39.6)	67 (60.4)	
Religion				
Yes	126 (21.2)	75 (59.5)	51 (40.5)	0.766
No	469 (78.8)	286 (61.0)	183 (39.0)	
Marital status				
Not married	48 (8.1)	26 (54.2)	22 (45.8)	0.145
Married	538 (90.4)	327 (60.8)	211 (39.2)	
Divorce/widow	9 (1.5)	8 (88.9)	1 (11.1)	
Profession				
Farmer-fisherman	248 (47.1)	156 (62.9)	92 (37.1)	**< 0.001**
Craftsmen	27 (4.5)	21 (77.8)	6 (22.2)	
Civil servant	27(4.5)	5 (18.5)	22 (81.5)	
Worker	42 (7.1)	15 (35.7)	27 (64.3)	
Business	63 (10.6)	35 (55.6)	28 (44.4)	
Building	67 (11.3)	39 (58.2)	28 (41.8)	
Housewife	60 (10.1)	41 (68.3)	19 (31.7)	
Older people	61 (10.3)	49 (80.3)	12 (19.7)	
Financial family status				
Poor household	39 (6.6)	31 (79.5)	8 (20.5)	**< 0.001**
Fairly poor households	59 (9.9)	50 (84.7)	9 (15.3)	
Other	497 (83.5)	280 (56.3)	217 (43.7)	
Self-assessment of current health status				
Satisfied	499 (83.9)	274 (54.9)	225 (45.1)	**< 0.001**
Unsatisfied	96 (16.1)	87 (90.6)	9 (9.4)	
Affected by flood from last year				
No	241 (40.5)	130 (53.9)	111 (46.1)	**0.012**
Disrupted	241 (40.5)	162 (67.2)	79 (32.8)	
Flooded	113 (19.0)	69 (61.1)	44 (38.9)	
Affected by storms from last year				
Yes	339 (57.0)	225 (66.4)	114 (33.6)	**0.001**
No	256 (43.0)	136 (53.1)	120 (46.9)	
Social support				
High	544 (91.4)	318 (58.5)	226 (41.5)	**< 0.001**
Low	51 (8.6)	43 (84.3)	8 (15.7)	
Family conflict				
Yes	47 (7.9)	39 (83.0)	8 (17.0)	**< 0.001**
No	548 (92.1)	322 (58.8)	226 (41.2)	

The numbers in the parentheses mean the percentages.

Significant values are in bold.

Table 1 also presents that the QoL of subjects was significantly affected by age, educational background, professions, financial family status, self-assessment of current health status, impact of storms and floods last year, family conflicts, and social support (*p* < 0.05).

### QoL of research participants

Table 2 shows the QoL of the study participants quantified using the WHOQOL-BREF scale. The overall score of QoL was 61.1 ± 10.8. The domain with the highest score was social relationships, at 63.4 ± 13.4, while the physical health domain received the lowest score of 57.2 ± 12.3. Except for social relationships, more than half of the people living in coastal areas did not have good QoL. The QoL score of the domains for people affected by flooding was significantly lower than that of those not affected, except for social relationships.

**Table 2:** Quality of life of research participants quantified using the WHOQOL-BREF scale by flood(n = 595).

Domains	Affected by Flooded (n = 354) (Mean score ± SD)	No flooded (n = 241) (Mean score ± SD)	General (n = 595) (Mean score ± SD)	General subjects with good QoL (%)
Physical health	55.6 ± 12.3	59.4 ± 12.0	57.2 ± 12.3*	25.2
Psychological health	60.3 ± 12.9	64.1 ± 12.8	61.9 ± 13.0*	42.0
Social relationships	63.4 ± 13.0	63.5 ± 14.0	63.4 ± 13.4	53.4
Environment	60.2 ± 14.0	64.4 ± 11.7	61.9 ± 13.3*	42.2
Overall evaluation	59.9 ± 10.9	62.9 ± 10.5	61.1 ± 10.8*	39.3

*Compared QoL (Mean score) of the subjects affected and non affected flood with p < 0.05 using Mann–Whitney U Test.

Table 3 shows the health issues reported by the participants and their associations with their QoL. Health problems with high prevalence at the study sites included musculoskeletal diseases (34.6%) and digestive disorders (21.7%). Respiratory, digestive, dermatological, and musculoskeletal disorders were identified as significant factors affecting QoL (*p* < 0.05).

**Table 3:** Health issues of the research subjects (n = 595) and the association quality of life. The numbers in the parentheses mean the percentages.

Diseases	All subjects (n = 595)		Overall *quality of life*				*p*
			Not good (n = 361)		Good (n = 234)		
	Suffered	Non-suffered	Suffered	Non-suffered	Suffered	Non-suffered	
Respiratory	66 (11.1)	529 (88.9)	49 (13.6)	312 (86.4)	17 (7.3)	217 (92.7)	**0.017**
Digestion	129 (21.7)	466 (78.3)	93 (25.8)	268 (74.2)	36 (15.4)	198 (84.6)	**0.003**
Dermatology	38 (6.4)	557 (93.6)	30 (8.3)	331 (91.7)	8 (3.4)	226 (96.6)	**0.017**
Allergy	19 (3.2)	576 (96.8)	14 (3.9)	347 (96.1)	5 (2.1)	229 (97.9)	0.238
Blood	16 (2.7)	579 (97.3)	9 (2.5)	352 (97.5)	7 (3.0)	227 (97.0)	0.714
Muscul-oskeletal	206 (34.6)	389 (65.4)	152 (42.1)	209 (57.9)	54 (23.1)	180 (76.9)	** < 0.001**

Significant values are in bold.

### Logistic regression model

Table 4 shows the results of the logistic regression analysis used to identify factors associated with not good QoL among the participants. Subjects with not good QoL had the educational background with no formal education (Odds ratio (OR) = 2.63, 95% CI 1.19–5.83), fairly poor/poor households (OR = 2.75, 95% CI 1.48–5.12), suffered Musculoskeletal diseases (OR = 1.61, 95%CI 1.02–2.56), unsatisfaction with health status (OR = 5.27, 95% CI 2.44–11.37), family conflicts (OR = 4.51, 95%CI 2.10–9.69), and low levels of social support (OR = 2.62; 95% CI 1.14–6.02). The analysis also revealed that workers (OR = 0.17, 95% CI 0.04–0.66) had a better QoL than farmer-fisherman. Among people affected by flood, factors associated with QoL included their profession, suffered musculoskeletal diseases, unsatisfaction with health status, and family conflicts.

**Table 4:** Results of logistic regression analysis to identify factors associated significantly with not good QoL of research subjects with all domains of QoL BREF.

Factors	General (n = 595)		Affected by Flooded (n = 354)		No flooded (n = 241)	
	Adjusted OR (CI 95%)	*p*	Adjusted OR (CI 95%)	*p*	Adjusted OR (CI 95%)	*p*
Age group						
18–29 (ref)	1	–	1	–	1	–
30–39	1.07 (0.49–2.36)	0.866	0.91 (0.29–2.86)	0.877	1.51 (0.44–5.29)	0.517
40–49	1.10 (0.57–2.14)	0.773	0.70 (0.29–1.72)	0.437	1.73 (0.57–5.26)	0.333
50–59	1.08 (0.57–2.05)	0.818	1.07 (0.45–2.58)	0.878	1.41 (0.51–3.94)	0.509
≥ 60	0.98 (0.58–1.64)	0.934	0.68 (0.34–1.36)	0.276	1.68 (0.71–4.00)	0.242
Educational background						
High school and above (ref)	1	–	1	–	1	–
No formal education	2.63 (1.19–5.83)	**0.017**	2.57 (0.91–7.24)	0.075	3.17 (0.79–12.82)	0.105
Primary school	1.65 (0.89–3.08)	0.111	1.47 (0.62–3.51)	0.384	1.95 (0.71–5.37)	0.198
Secondary school	1.18 (0.65–2.15)	0.583	1.43 (0.62–3.28)	0.398	1.04 (0.39–2.79)	0.943
Profession						
Farmer-fisherman (ref)	1	–	1	–	1	–
Craftsman	0.86 (0.38–1.91)	0.703	0.75 (0.25–2.24)	0.607	0.76 (0.20–2.86)	0.683
Civil servant	1.87 (0.53–6.58)	0.330	0.65 (0.11–3.96)	0.637	3.26 (0.42–25.12)	0.257
Worker	**0.17 (0.04–0.66)**	**0.01**	**0.15 (0.03–0.86)**	**0.033**	0.18 (0.02–2.04)	0.166
Business	0.49 (0.17–1.46)	0.203	0.66 (0.12–3.57)	0.633	0.35 (0.07–1.80)	0.206
Building	0.65 (0.25–1.69)	0.380	0.64 (0.17–2.42)	0.514	0.61 (0.13–2.76)	0.516
Housewife	0.94 (0.36–2.46)	0.904	1.27 (0.34–4.81)	0.725	0.64 (0.14–2.97)	0.566
Older people	0.96 (0.36–2.53)	0.936	0.84 (0.20–3.54)	0.813	1.13 (0.26–4.95)	0.876
Financial family status						
Other (ref)	1	–	1	–	1	–
Fairly poor/poor households	2.75 (1.48–5.12)	**0.001**	1.43 (0.61–3.40)	0.413	**5.43 (2.06–14.3)**	**0.001**
Respiratory diseases						
Yes (ref)	1	–	1	–	1	–
No	1.40 (0.71–2.77)	0.327	1.72 (0.72–4.11)	0.223	0.76 (0.22–2.63)	0.660
Digestion diseases						
Yes (ref)	1	–	1	–	1	–
No	1.11 (0.67–1.84)	0.686	1.84 (0.93–3.66)	0.082	0.49 (0.20–1.25)	0.136
Dermatology diseases						
Yes (ref)	1	–	1	–	1	–
No	2.07 (0.83–5.17)	0.117	1.54 (0.55–4.31)	0.408	7.28 (0.62–85.44)	0.114
Musculoskeletal diseases						
No (ref)	1	–	1	–	1	–
Yes	1.61 (1.02–2.56)	**0.041**	**2.56 (1.38–4.74)**	**0.003**	0.86 (0.38–1.92)	0.708
Self-assessment of current health status						
Satisfied (ref)	1	–	1	–	1	–
Unsatisfied	**5.27 (2.44–11.37)**	**< 0.001**	**4.21 (1.57–11.28)**	**0.004**	**5.56 (1.42–21.74)**	**0.014**
Family conflicts						
Yes (ref)	**4.51 (2.10–9.69)**	** < 0.001**	6.40 (2.40–17.08)	** < 0.001**	**3.90 (1.00–15.23)**	**0.05**
No	1	–	1	–	1	–
Social support						
Low	2.62 (1.14–6.02)	**0.023**	7.15 (0.88–58.26)	0.066	2.28 (0.81–6.43)	0.120
High (ref)	1		1	–	1	–

Ref, Reference; OR, Odds ratio.

Significant values are in bold.

## Discussion

The present study assessed the QoL and its associated factors among people living in coastal communes in central Vietnam. The findings indicated that 39.3% of research subjects had good QoL, especially those with a low score in the physical health domain. QoL was influenced by educational background, profession, family financial status, musculoskeletal diseases, self-assessment of current health, family conflicts, and social support. To the best of our knowledge, this is the first study to use the WHOQOL-BREF to measure the QoL in the general population of Southeast Asia.

The overall QoL score (61.1 ± 10.8) living in coastal areas was higher than that of people living near solid waste management facilities in Vietnam^14^ but lower than the global average^15^. The QoL of the target population in this study was lower than that for the Pakistani and Indonesian populations in all four domains, except environment^16,17^. The low QoL scores obtained in this study could be attributed to unsatisfactory living conditions, access to healthcare, transportation, quality education, security, physical mobility, entertainment, and shopping centers in coastal communes^17^.

The lowest score was in the physical health domain (57.2 ± 12.3), indicating unhealthy surroundings. Unhealthy surroundings can adversely affect health status^16^. Meanwhile, the highest score in the social relationship domain (63.4 ± 13.4), which was also observed in Pakistan^17^. This was probably due to the neighborly relationship creating a strong connection between individuals and communities in coastal areas. This finding is supported by a previous study that revealed a significant positive relationship between social cohesion and QoL^18^. Through living in an area for generations, people become familiar with their neighbors and who can receive assistance for major life events, such as marriage and illness. Interestingly, the QoL score of the domains for people affected by flooding was significantly lower than that of those not affected, except for social relationships. Our findings support previous studies that have confirmed the detrimental effects of flooding on QoL^19–23^. Therefore, to lessen the affects of flooding, it is crucial to offer residents in flood-affected areas psychological counseling as well as physical and environmental supports.

Subjects who were no formal education were likely to have lower QoL than those with high school education and above (OR = 2.63). This finding is supported by previous studies^24,25^, which reported that lower educational levels were related to unhappiness and poor social relationships.

Profession was significantly associated with QoL. This result is consistent with previous studies reporting significant impacts of severe workload, economic categorization, and job pressure^26–28^. The stability of workload and income, both of which are closely related to the job, also impact QoL. Reducing work intensity is expected to improve the QoL.

Family financial status is also associated with QoL. People living in low-income households were 2.75 times more likely to have a significantly lower QoL than those living in higher-income households. Family financial was reported to be associated with all domains of QoL except the physical domain (Appendix 2–5). This is consistent with a previous study by Rajput et al.^29^, who argued that the higher socioeconomic status of the study participants helps them have a better QoL .

Although some diseases, such as respiratory, digestive, and dermatological diseases, were identified as significant factors by univariate logistic regression analysis, multivariable logistic regression analysis found that these factors were not associated with QoL, except for musculoskeletal diseases. Previous studies have reported that diseases affect QoL^30–32^, necessitating further longitudinal studies to confirm the results obtained for this population. More comprehensively, the present study revealed that people who were dissatisfied with their health were 5.14 times more likely to have a lower QoL than others. Moreover, unsatisfaction with health status was found to be strongly associated with all domains of QoL (Appendix 2–5). This is consistent with previous studies in Iran and Norway, demonstrating that self-reported health status was the most substantial factor for QoL^33,34^. Another study argued that poor health status has a negative impact on QoL^14^, recommending a revision of public health policies in the study areas.

Family environment and social support significantly affected QoL. Frequent conflicts with their families decreased the QoL of the participants (OR = 4.25), similar to a study in Malaysia that reported that work-family conflict was associated with QoL^35^. Besides, with the exception of the psychological domain, family conflict was found to be related to all QoL domains (Appendix 2–5). A low level of social support decreased QoL (OR = 2.56). Social support, which has the potential to improve QoL in target communities, has been reported as a QoL predictor in previous studies^35,36^.

### Implications and future study

Considering the low level of physical health found in this study, the priority for countermeasures should be to improve this domain. Future studies should follow up on the QoL after countermeasures are implemented. In addition, the QoL obtained in this study should be compared with those of different areas, which allowed us to better understand the factors associated with QoL. Findings from such studies, as well as those from the present study, will help governments and local authorities develop policies pertaining to residents of unhealthy communities, such as coastal communes.

### Strengths and limitations

The main limitation of this cross-sectional study was the difficulty in investigating QoL and its related factors over a long period, although QoL is highly variable over time. For example, the temporal change in QoL after a flood event, which would gradually recover, could not be analyzed in this study.

One of the advantages of this study was the use of a validated and standardized WHOQOL-BREF scale, which enabled a comparison of the obtained QoL with other reports. Another advantage was the analysis of community-based QoL, especially focusing on healthy people living in coastal areas, whereas most previous studies analyzed QoL only in diseased and handicapped populations. This study contributes to a better understanding of the QoL of people in monsoon Asia affected by frequent floods and storms, which has been poorly investigated.

## Conclusion

To the best of our knowledge, this was the first study to analyze QoL and its association with sociodemographic variables and the impact of floods on the general Vietnamese population. Overall, the QoL of residents in disadvantaged communes in coastal areas was low, with only 39.3% of the participants having a good QoL. Among the four domains of the WHOQOL-BREF scale, the physical health domain showed the lowest score (57.2 ± 12.3), while the social participation domain had the highest score (63.4 ± 13.4). The QoL score in all domains was notably lower for individuals impacted by flooding compared to those who were unaffected, with the exception of social relationships. Farmer-fishermen, low income, musculoskeletal diseases, dissatisfaction with their current health status, family conflicts, and less social support contributed to lower QoL.

QoL, especially in terms of physical health, in the general population has not received much attention. This study demonstrated that a challenging economic climate, inadequate medical facilities and services, and the risk of numerous natural disasters, such as floods, are contributing factors to the lower QoL. Local authorities need to take more appropriate and practical measures to increase their support, including all aspects of physical and mental health, social relations, and living environments, to improve the QoL of people living in these problematic communes.

## Methods

### Study design and setting

A cross-sectional descriptive study was conducted in coastal communes with disadvantages as described below in Thua Thien Hue province, central Vietnam. This province has a tropical monsoon climate with 3000 mm of annual rainfall on average. Floods generally begin in October, followed by the rainy season in September. According to the Decision of the Prime Minister of Vietnam, Thua Thien Hue Province has seven communes that have been approved as poor communes with particular challenges in the lowlands, coastal areas, and islands of Vietnam for the period of 2021–2025. These seven communes have an approximate population of 45,000 and a rate of poor and fairly poor households of 15% or more or are affected by salinity intrusion continuously for three months or more during the year and have a rate of poor and fairly poor households (e.g. income of 1,500,000 VND (~ 61 USD)/person/month and lack of basic social services including employment, health, education, housing, water and sanitation, and information) of 12% or more^37,38^.

This study employed the following multistage sampling method: Two of the seven poor communes were selected randomly: Giang Hai in Phu Loc district and Phu Gia in Phu Vang district. From the selected communes, four villages were randomly selected. The chosen villages included Giang Che village and Nam Truong village in the Giang Hai commune and Ha Tru Thuong and Mong B villages in the Phu Gia commune.

### Subjects

The required study sample size was calculated as follows:

$$ {\text{n}} = {\text{Z}}^{{2}}_{{({1} - \alpha /{2})}} \frac{{\left( {1 - p} \right)p}}{{d^{2} }}^{39} $$

^39^ where p was set at 0.50 because the proportion of subjects with good QoL was unknown^40^, d was set at 0.05 as a desired error^40^, and Z_1−α/2_ for reliability was set at 1.95 with 95% confidence (alpha = 0.05). This equation set the minimum sample size at 384. A larger sample (n = 595) was selected based on the following criteria: (a) 18 years old or older, (b) present during the study period, (c) lived continuously at the study sites for at least 12 months before the study, and (d) willing to participate in the study. Patients with mental health problems were excluded.

### Data collection

Data were collected from October 2022 to February 2023 by students at the University of Medicine and Pharmacy, Hue University, using questionnaires prepared based on in-person interviews with research participants. All students had studied preventive medicine and received comprehensive training before data collection.

To determine the factors influencing QoL, general characteristics of the participants, including gender, age, educational background (no formal education/primary school/secondary school/high school and above), religion, marital status (not married/married/divorced/widow), occupation, financial family status (poor/fairly poor/other), smoking status, alcohol consumption in the last 30 days, self-assessment of current health status, impact of storms in the previous year, and family conflict, were obtained through face-to-face interviews.

We asked the respondents about the impact of floods in the previous year and obtained answers of no, disrupted, or flooded. The disrupted subjects were those who did not have floodwater in the habitable spaces of their homes but faced interruption as a result of floods. For example, participants that experienced flooding in non-habitable spaces and were disrupted by the loss of utilities and limited access to services. On the other hand, people whose homes had at least one habitable room (e.g. a living room, kitchen, or bedroom) with flooding were defined as the flooded subjects^19^.

Social support was assessed using a Multidimensional Scale of Perceived Social Support^41^. Twelve questions were asked to assess participants' sense of support from friends, family, and significant others. Although the original scale uses a 7-point Likert-type scale, our questionnaire reduced it to a 5-point scale including strongly disagree (= 1), disagree (= 2), neutral (= 3), agree (= 4), and strongly agree (= 5), according to a previous study in Vietnam^42^, to identify subjects with low social support defined as a mean score of less than three.

QoL, as the dependent variable, was assessed using the WHO's QoL assessment scale (WHOQOL-BREF)^43^. Many researchers have used this scale to assess QoL in Vietnam and worldwide, focusing on different topics under various circumstances^44–48^. In many countries, the WHOQOL-BREF is regarded as extraordinarily trustworthy and culturally appropriate for assessing QoL and might be helpful in studies requiring quick evaluation of QoL^49,50^. As this method can examine individual views in the context of culture, personal objectives, standards, and concerns, it has been widely field-tested and validated^14^.

### Data analysis

QoL was quantified based on 26 questions from four main domains: physical health, psychology, environment, and social relationships, with a relatively high consistency (Cronbach's alpha > 0.7). These facets are assigned scores ranging from extremely bad (= 1) to very good (= 5).

Based on this Likert scale, we employed a specific formula to determine the score for each domain (Appendix 1). The QoL score was derived by averaging the scores of the four aforementioned areas. The QoL was assessed based on the obtained score, with a higher score indicating better QoL. The following criteria were used: those with scores of less than 33.3, 33.3–66.7, and greater than 66.7% were considered to have poor, average, and good QoL, respectively. In this study, subjects with a score higher than 66.7% had good QoL, while the others had not good QoL^14,51^.

SPSS Statistics for Windows (version 20.0). Armonk, NY: IBM Corp.) was used for data analysis. Compared the means of QoL score between study groups with Mann–Whitney U Test. Multivariable logistic regression was performed to determine the factors related to the QoL of residents living in coastal areas. Independent variables with statistical significance in the univariate logistic regression analysis were selected for multivariate logistic regression. A *p* < 0.05 was considered statistically.

### Institutional review board statement

The study was conducted in accordance with the Declaration of Helsinki, and approved by the Ethical Committee in Biomedical Research of the University of Medicine and Pharmacy, Hue University (Code: H2022/486, dated June 30, 2022). The study was also approved by local authorities in the areas where the study was conducted. The participants willingly participated after being informed of the study's goals and topics. These data were only used for analysis, providing findings for the better health of individuals.

### Informed consent

Informed consent was obtained from all participants involved in the study. For participants who were no formal education, written informed consents were obtained from their legal guardians.

### Supplementary Information

Supplementary Tables.

## Data Availability

The datasets generated during and/or analyzed during this study are not publicly available but are available from the corresponding author on reasonable request.
