# Supplementary material for: Determining the factors impacting the quality of life among the general population in coastal communities in central Vietnam
Source: Sci Rep. 2024 Mar 24;14:6986. doi: 10.1038/s41598-024-57672-0 (PMC10961306; doi:10.1038/s41598-024-57672-0)
Supplement: Supplementary file 1 — Supplementary Tables. [file 41598_2024_57672_MOESM1_ESM.docx]

**APPENDIX 1: Calculation of quality of life score**

1) In each domain, the mean value (M) of scores (1 to 5) corresponding to answers to questions is calculated. Then, the domain mean score (D) is calculated by the following formula:

D = (M x 4 – 4) x (100/16).

2) The overall evaluation is made with the score determined by averaging the four domain mean scores.

3) Table S1 is the example of calculation of quality of life score for a subject.

Table S1. Calculation of quality of life score for Subject #10.

| **Subject #51** | **Score of answer to each question** | | | | |
| --- | --- | --- | --- | --- | --- |
|  | **1** | **2** | **3** | **4** | **5** |
| 1. **Physical health** |  |  |  |  |  |
| Q.1.1 |  |  | ✓ |  |  |
| Q.1.2 |  |  | ✓ |  |  |
| Q.1.3 |  |  | ✓ |  |  |
| Q.1.4 | ✓ |  |  |  |  |
| Q.1.5 |  |  |  | ✓ |  |
| Q.1.6 |  |  | ✓ |  |  |
| Q.1.7 |  |  | ✓ |  |  |
| Domain mean score | $(\frac{20}{7}\times4-4)\times(\frac{100}{16})=46.43$ | | | | |
| 1. **Psychological health** |  |  |  |  |  |
| Q.2.1 |  |  |  | ✓ |  |
| Q.2.2 |  |  | ✓ |  |  |
| Q.2.3 |  | ✓ |  |  |  |
| Q.2.4 |  | ✓ |  |  |  |
| Q.2.5 |  |  | ✓ |  |  |
| Q.2.6 |  |  |  | ✓ |  |
| Domain mean score | $(\frac{18}{6}\times4-4)\times(\frac{100}{16})=50.00$ | | | | |
| 1. **Social relationship** |  |  |  |  |  |
| Q.3.1 |  |  |  | ✓ |  |
| Q.3.2 |  |  |  |  | ✓ |
| Q.3.3 |  |  | ✓ |  |  |
| Domain mean score | $(\frac{12}{3}\times4-4)\times(\frac{100}{16})=75.00$ | | | | |
| 1. **Environment** |  |  |  |  |  |
| Q.4.1 | ✓ |  |  |  |  |
| Q.4.2 |  |  |  |  | ✓ |
| Q.4.3 |  |  | ✓ |  |  |
| Q.4.4 | ✓ |  |  |  |  |
| Q.4.5 |  | ✓ |  |  |  |
| Q.4.6 |  |  |  | ✓ |  |
| Q.4.7 |  |  | ✓ |  |  |
| Q.4.8 |  |  | ✓ |  |  |
| Domain mean score | $(\frac{22}{8}\times4-4)\times(\frac{100}{16})=43.75$ | | | | |
| **Overall evaluation** | $\frac{46.43 + 50.00+ 75.00 + 43.75}{4}=53.79$ | | | | |

**Appendix 2. Results of logistic regression analysis to identify factors associated significantly with not good QoL of research subjects with Physical domain of QoL BREF**

| **Factors** | | **General (n=595)** | | **Affected by Flooded**  **(n=354)** | | **No flooded (n=241)** | |
| --- | --- | --- | --- | --- | --- | --- | --- |
|  | | **Adjusted OR**  **(CI 95%)** | ***p*** | **Adjusted OR**  **(CI 95%)** | ***p*** | **Adjusted OR**  **(CI 95%)** | ***p*** |
| Age group | 18–29 (ref) | 1 | - | 1 | - | 1 | - |
|  | 30–39 | 1.56 (0.69–3.54) | 0.291 | 1.11 (0.32–3.81) | 0.871 | 1.61 (0.47–5.50) | 0.449 |
|  | 40–49 | 1.76 (0.89–3.49) | 0.103 | 1.96 (0.74–5.20) | 0.176 | 2.29 (0.77–6.84) | 0.137 |
|  | 50–59 | 1.52 (0.79–2.94) | 0.213 | 1.43 (0.57–3.62) | 0.446 | 1.51 (0.53–4.29) | 0.442 |
|  | ≥ 60 | 0.76 (0.41–1.39) | 0.370 | 0.79 (0.34–1.81) | 0.574 | 0.51 (0.20–1.31) | 0.159 |
| Educational background | High school and above (ref) | 1 | - | 1 | - | 1 | - |
|  | No formal education | 0.49 (0.20–1.21) | 0.121 | 0.28 (0.08–0.94) | **0.039** | 0.65 (0.14–3.10) | 0.585 |
|  | Primary school | 1.16 (0.60–2.22) | 0.659 | 0.65 (0.26–1.66) | 0.369 | 2.06 (0.73–5.81) | 0.173 |
|  | Secondary school | 0.98 (0.52–1.84) | 0.943 | 0.65 (0.26–1.62) | 0.356 | 1.14 (0.42–3.08) | 0.797 |
| Profession | Farmer-fisherman (ref) | 1 | - | 1 | - | 1 | - |
|  | Craftsman | 2.13 (0.75–6.03) | 0.153 | 1.82 (0.47–7.14) | 0.389 | 5.05 (0.89–28.58) | 0.067 |
|  | Civil servant | 1.21 (0.29–4.99) | 0.794 | 1.77 (0.24–13.42) | 0.579 | 1.11 (0.12–10.62) | 0.928 |
|  | Worker | 2.17 (0.54–8.79) | 0.278 | 1.38 (0.23–8.45) | 0.725 | 4.04 (0.38–43.45) | 0.249 |
|  | Business | 4.94 (1.40–17.42) | **0.013** | 1.94 (0.30–12.63) | 0.486 | 12.70 (1.74–92.84) | **0.012** |
|  | Building | 2.44 (0.76–7.83) | 0.133 | 3.80 (0.81–17.75) | 0.090 | 1.82 (0.25–13.38) | 0.555 |
|  | Housewife | 1.23 (0.37–4.11) | 0.738 | 0.74 (0.14–3.92) | 0.718 | 3.06 (0.44–21.37) | 0.259 |
|  | Older people | 1.52 (0.45–5.10) | 0.498 | 0.78 (0.13–4.89) | 0.794 | 2.65 (0.39–17.77) | 0.317 |
| Financial family status | Other (ref) | 1 | - | 1 | - | 1 | - |
|  | Fairly poor/poor households | 0.97 (0.53–1.79) | 0.932 | 1.45 (0.59–3.54) | 0.417 | 0.66 (0.27–1.60) | 0.354 |
| Respiratory diseases | Yes (ref) | 1 | - | 1 | - | 1 | - |
|  | No | 1.67 (0.86–3.24) | 0.131 | 1.54 (0.66–3.61) | 0.319 | 2.48 (0.58–10.54) | 0.220 |
| Digestion diseases | Yes (ref) | 1 | - | 1 | - | 1 | - |
|  | No | 1.25 (0.72–2.17) | 0.422 | 1.43 (0.69–2.98) | 0.335 | 1.76 (0.67–4.65) | 0.252 |
| Dermatology diseases | Yes (ref) | 1 | - | 1 | - | 1 | - |
|  | No | 0.60 (0.23–1.55) | 0.290 | 1.03 (0.36–2.94) | 0.960 | 0.000 (0.000–0.000) | 0.999 |
| Musculoskeletal diseases | No (ref) | 1 | - | 1 | - | 1 | - |
|  | Yes | 0.50 (0.29–0.85) | **0.010** | 0.43 (0.21–0.88) | **0.020** | 0.55 (0.22–1.38) | 0.200 |
| Self-assessment of current health status | Unsatisfied (ref) | 1 | - | 1 | - | 1 | - |
|  | Satisfied | 0.39 (0.18–0.86) | **0.019** | 0.41 (0.15–1.16) | 0.092 | 0.50 (0.12–1.99) | 0.322 |
| Family conflicts | Yes (ref) | 0.38 (0.18–0.77) | **0.008** | 0.25 (0.10–0.63) | **0.003** | 0.73 (0.20–2.62) | 0.626 |
|  | No | 1 | – | 1 | - | 1 | - |
| Social support | Low | 1.41 (0.68–2.94) | 0.354 | 0.18 (0.02–1.51) | 0.113 | 3.09 (1.17–8.14) | **0.022** |
|  | High (ref) | 1 |  | 1 | - | 1 | - |

**Appendix 3. Results of logistic regression analysis to identify factors associated significantly with not good QoL of research subjects with Psychological domain of QoL BREF**

| **Factors** | | **General (n=595)** | | **Affected by Flooded**  **(n=354)** | | **No flooded (n=241)** | |
| --- | --- | --- | --- | --- | --- | --- | --- |
|  | | **Adjusted OR**  **(CI 95%)** | ***p*** | **Adjusted OR**  **(CI 95%)** | ***p*** | **Adjusted OR**  **(CI 95%)** | ***p*** |
| Age group | 18–29 (ref) | 1 | - | 1 | - | 1 | - |
|  | 30–39 | 1.87 (0.85–4.09) | 0.118 | 2.69 (0.87–8.31) | 0.085 | 1.26 (0.38–4.18) | 0.709 |
|  | 40–49 | 1.01 (0.52–1.94) | 0.981 | 1.19 (0.48–2.93) | 0.703 | 0.89 (0.31–2.54) | 0.828 |
|  | 50–59 | 1.15 (0.61–2.16) | 0.669 | 0.66 (0.27–1.66) | 0.382 | 1.94 (0.70–5.40) | 0.204 |
|  | ≥ 60 | 1.51 (0.91–2.53) | 0.114 | 1.83 (0.92–3.64) | 0.083 | 1.20 (0.54–2.79) | 0.677 |
| Educational background | High school and above (ref) | 1 | - | 1 | - | 1 | - |
|  | No formal education | 0.28 (0.13–0.63) | **0.002** | **0.21 (0.07**–0.60) | **0.004** | 0.45 (0.12–1.68) | 0.234 |
|  | Primary school | 0.58 (0.31–1.08) | 0.085 | 0.53 (0.23–1.25) | 0.149 | 0.68 (0.26–1.79) | 0.432 |
|  | Secondary school | 0.64 (0.35–1.16) | 0.138 | 0.43 (0.19–0.98) | **0.045** | 0.92 (0.35–2.37) | 0.857 |
| Profession | Farmer-fisherman (ref) | 1 | - | 1 | - | 1 | - |
|  | Craftsman | 0.62 (0.29–1.35) | 0.228 | 0.91 (0.31–2.62) | 0.855 | 0.42 (0.11–1.57) | 0.196 |
|  | Civil servant | 0.45 (0.14–1.42) | 0.174 | 0.97 (0.18–5.37) | 0.972 | 0.24 (0.04–1.41) | 0.115 |
|  | Worker | 3.50 (0.84–14.58) | 0.086 | 6.86 (1.02–46.38) | **0.048** | 1.65 (0.14–19.51) | 0.693 |
|  | Business | 0.92 (0.32–2.65) | 0.880 | 1.01 (0.19–5.23) | 0.994 | 0.78 (0.16–3.93) | 0.766 |
|  | Building | 0.99 (0.39–2.50) | 0.983 | 1.91 (0.53–6.94) | 0.320 | 0.47 (0.10–2.15) | 0.327 |
|  | Housewife | 0.45 (0.17–1.15) | 0.093 | 0.45 (0.12–1.71) | 0.242 | 0.38 (0.08–1.77) | 0.216 |
|  | Older people | 0.52 (0.20–1.34) | 0.174 | 0.81 (0.19–3.39) | 0.767 | 0.27 (0.06–1.20) | 0.085 |
| Financial family status | Other (ref) | 1 | - | 1 | - | 1 | - |
|  | Fairly poor/poor households | 0.37 (0.20–0.67) | **0.001** | 0.41 (0.16–1.05) | 0.063 | 0.32 (0.14–0.73) | **0.007** |
| Respiratory diseases | Yes (ref) | 1 | - | 1 | - | 1 | - |
|  | No | 0.67 (0.35–1.30) | 0.238 | 0.63 (0.27–1.49) | 0.295 | 0.54 (0.16–1.83) | 0.324 |
| Digestion diseases | Yes (ref) | 1 | - | 1 | - | 1 | - |
|  | No | 0.96 (0.58–1.58) | 0.867 | 0.79 (0.41–1.53) | 0.489 | 1.56 (0.62–3.95) | 0.346 |
| Dermatology diseases | Yes (ref) | 1 | - | 1 | - | 1 | - |
|  | No | 0.64 (0.28–1.48) | 0.297 | 0.94 (0.36–2.48) | 0.907 | 0.10 (0.01–1.09) | 0.059 |
| Musculoskeletal diseases | No (ref) | 1 | - | 1 | - | 1 | - |
|  | Yes | 0.69 (0.44–1.07) | 0.098 | 0.52 (0.29–0.95) | **0.033** | 1.05 (0.48–2.31) | 0.902 |
| Self-assessment of current health status | Unsatisfied (ref) | 1 | - | 1 | - | 1 | - |
|  | Satisfied | 0.12 (0.05–0.28) | **<0.001** | 0.22 (0.09–0.59) | **0.002** | 0.04 (0.00–0.28) | **0.002** |
| Family conflicts | Yes (ref) | 0.68 (0.33–1.38) | 0.284 | 0.52 (0.21–1.34) | 0.179 | 0.66 (0.20–2.26) | 0.510 |
|  | No | 1 | – | 1 | - | 1 | - |
| Social support | Low | 0.87 (0.43–1.74) | 0.685 | 0.35 (0.07–1.68) | 0.190 | 1.10 (0.44–2.76) | 0.833 |
|  | High (ref) | 1 |  | 1 | - | 1 | - |

**Appendix 4. Results of logistic regression analysis to identify factors associated significantly with not good QoL of research subjects with Social Relationship domain of QoL BREF**

| **Factors** | | **General (n=595)** | | **Affected by Flooded**  **(n=354)** | | **No flooded (n=241)** | |
| --- | --- | --- | --- | --- | --- | --- | --- |
|  | | **Adjusted OR**  **(CI 95%)** | ***p*** | **Adjusted OR**  **(CI 95%)** | ***p*** | **Adjusted OR**  **(CI 95%)** | ***p*** |
| Age group | 18–29 (ref) | 1 | - | 1 | - | 1 | - |
|  | 30–39 | 1.75 (0.80–3.83) | 0.161 | 4.97 (1.49–16.52) | **0.009** | 0.63 (0.18–2.12) | 0.450 |
|  | 40–49 | 3.26 (1.59–6.69) | **0.001** | 9.85 (3.12–31.22) | **<0.001** | 1.36 (0.41–4.20) | 0.597 |
|  | 50–59 | 1.15 (0.63–2.12) | 0.644 | 1.03 (0.45–2.34) | 0.951 | 0.95 (0.34–2.65) | 0.926 |
|  | ≥ 60 | 0.92 (0.56–1.50) | 0.724 | 1.03 (0.54–1.95) | 0.933 | 0.68 (0.29–1.61) | 0.376 |
| Educational background | High school and above (ref) | 1 | - | 1 | - | 1 | - |
|  | No formal education | 0.87 (0.44–1.82) | 0.703 | 1.20 (0.46–3.12) | 0.708 | 0.48 (0.12–1.94) | 0.305 |
|  | Primary school | 1.09 (0.58–2.05) | 0.782 | 1.24 (0.52–2.96) | 0.633 | 1.04 (0.37–2.93) | 0.941 |
|  | Secondary school | 1.36 (0.73–2.53) | 0.335 | 1.17 (0.50–2.74) | 0.719 | 1.80 (0.64–5.07) | 0.267 |
| Profession | Farmer-fisherman (ref) | 1 | - | 1 | - | 1 | - |
|  | Craftsman | 1.02 (0.54–2.02) | 0.959 | 0.78 (0.34–1.82) | 0.568 | 1.80 (0.48–6.78) | 0.386 |
|  | Civil servant | 0.63 (0.21–1.89) | 0.409 | 0.44 (0.09–2.30) | 0.333 | 1.55 (0.27–9.01) | 0.627 |
|  | Worker | 5.24 (1.20–22.91) | **0.028** | 6.79 (0.70–65.86) | 0.099 | 5.53 (0.52–58.54) | 0.156 |
|  | Business | 1.57 (0.53–4.62) | 0.413 | 0.99 (1.19–0.15) | 0.986 | 5.46 (0.97–30.91) | 0.055 |
|  | Building | 1.65 (0.70–3.87) | 0.254 | 1.37 (0.45–4.22) | 0.582 | 2.72 (0.60–12.36) | 0.195 |
|  | Housewife | 0.57 (0.24–1.36) | 0.204 | 0.16 (0.05–0.54) | **0.003** | 2.69 (0.57–12.75) | 0.212 |
|  | Older people | 0.98 (0.42–0.31) | 0.971 | 0.39 (0.11–1.34) | 0.135 | 2.60 (0.59–11.52) | 0.207 |
| Financial family status | Other (ref) | 1 | - | 1 | - | 1 | - |
|  | Fairly poor/poor households | 0.32 (0.18–0.54) | **<0.001** | 0.48 (0.23–1.00) | 0.051 | 0.23 (0.10–0.54) | **0.001** |
| Respiratory diseases | Yes (ref) | 1 | - | 1 | - | 1 | - |
|  | No | 0.63 (0.35–1.14) | 0.125 | 0.60 (0.28–1.25) | 0.173 | 0.48 (0.14–1.63) | 0.238 |
| Digestion diseases | Yes (ref) | 1 | - | 1 | - | 1 | - |
|  | No | 1.22 (0.76–1.96) | 0.412 | 1.24 (0.68–2.26) | 0.484 | 1.28 (0.51–3.23) | 0.601 |
| Dermatology diseases | Yes (ref) | 1 | - | 1 | - | 1 | - |
|  | No | 1.04 (0.47–2.30) | 0.930 | 0.72 (0.28–1.82) | 0.486 | 1.82 (2.52–13.10) | 0.554 |
| Musculoskeletal diseases | No (ref) | 1 | - | 1 | - | 1 | - |
|  | Yes | 0.66 (0.43–1.00) | 0.052 | 0.43 (0.25–0.75) | **0.003** | 1.16 (0.52–2.62) | 0.714 |
| Self-assessment of current health status | Unsatisfied (ref) | 1 | - | 1 | - | 1 | - |
|  | Satisfied | 0.54 (0.32–0.93) | **0.027** | 0.89 (0.45–1.74) | 0.730 | 0.21 (0.07–0.69) | **0.010** |
| Family conflicts | Yes (ref) | 0.38 (0.18–0.80) | **0.012** | 0.29 (0.11–0.75) | **0.011** | 0.42 (0.10–1.71) | 0.225 |
|  | No | 1 | – | 1 | - | 1 | - |
| Social support | Low | 0.19 (0.09–0.42 | **<0.001** | 0.02 (0.0**0**–0.25) | **0.002** | 0.33 (0.13–0.86) | **0.023** |
|  | High (ref) | 1 |  | 1 | - | 1 | - |

**Appendix 5. Results of logistic regression analysis to identify factors associated significantly with not good QoL of research subjects with Environmental domain of QoL BREF**

| **Factors** | | **General (n=595)** | | **Affected by Flooded**  **(n=354)** | | **No flooded (n=241)** | |
| --- | --- | --- | --- | --- | --- | --- | --- |
|  | | **Adjusted OR**  **(CI 95%)** | ***p*** | **Adjusted OR**  **(CI 95%)** | ***p*** | **Adjusted OR**  **(CI 95%)** | ***p*** |
| Age group | 18–29 (ref) | 1 | - | 1 | - | 1 | - |
|  | 30–39 | 1.29 (0.59–2.83) | 0.523 | 1.01 (0.32–3.16) | 0.989 | 1.95 (0.56–6.77) | 0.294 |
|  | 40–49 | 1.39 (0.72–2.70) | 0.325 | 1.84 (0.74–4.61) | 0.192 | 1.20 (0.40–3.59) | 0.750 |
|  | 50–59 | 1.15 (0.61–2.16) | 0.673 | 0.90 (0.37–2.16) | 0.810 | 1.11 (0.39–3.17) | 0.842 |
|  | ≥ 60 | 1.03 (0.62–1.72) | 0.917 | 1.33 (0.67–2.65) | 0.423 | 0.70 (0.29–1.73) | 0.441 |
| Educational background | High school and above (ref) | 1 | - | 1 | - | 1 | - |
|  | No formal education | 0.34 (0.16–0.76) | **0.008** | 0.31 (0.11–0.87) | **0.025** | 0.34 (0.08–1.39) | 0.133 |
|  | Primary school | 0.58 (0.31–1.07) | 0.082 | 0.58 (0.24–1.29) | 0.220 | 0.64 (0.23–1.75) | 0.382 |
|  | Secondary school | 0.70 (0.38–1.28) | 0.245 | 0.44 (0.19–1.03) | 0.058 | 1.26 (0.47–3.38) | 0.650 |
| Profession | Farmer-fisherman (ref) | 1 | - | 1 | - | 1 | - |
|  | Craftsman | 1.00 (0.46–2.19) | 0.994 | 1.02 (0.36–2.90) | 0.965 | 1.52 (0.36–6.40) | 0.572 |
|  | Civil servant | 0.64 (0.20–2.06) | 0.451 | 1.16 (0.19–6.92) | 0.873 | 0.74 (0.11–4.93) | 0.757 |
|  | Worker | 5.44 (1.27–23.30) | **0.022** | 6.53 (0.95–44.78) | 0.056 | 5.52 (0.45–68.02) | 0.182 |
|  | Business | 1.33 (0.45–3.92) | 0.605 | 0.88 (0.16–4.79) | 0.882 | 2.35 (0.41–13.45) | 0.338 |
|  | Building | 1.37 (0.53–3.50) | 0.514 | 1.34 (0.37–4.86) | 0.653 | 1.96 (0.39–9.73) | 0.412 |
|  | Housewife | 0.73 (0.28–1.89) | 0.512 | 0.51 (0.14–1.89) | 0.315 | 1.22 (0.23–6.35) | 0.814 |
|  | Older people | 0.74 (0.28–1.92) | 0.531 | 0.74 (1.18–3.07) | 0.681 | 0.76 (0.16–3.75) | 0.737 |
| Financial family status | Other (ref) | 1 | - | 1 | - | 1 | - |
|  | Fairly poor/poor households | 0.23 (0.12–0.43) | **<0.001** | 0.38 (0.15–0.95) | **0.038** | 0.11 (0.04–0.33) | **<0.001** |
| Respiratory diseases | Yes (ref) | 1 | - | 1 | - | 1 | - |
|  | No | 0.82 (0.43–1.57) | 0.547 | 0.57 (0.24–1.36) | 0.207 | 2.16 (0.60–7.76) | 0.238 |
| Digestion diseases | Yes (ref) | 1 | - | 1 | - | 1 | - |
|  | No | 0.79 (0.48–1.31) | 0.362 | 0.41 (0.20–0.81) | **0.011** | 2.53 (0.97–6.60) | 0.059 |
| Dermatology diseases | Yes (ref) | 1 | - | 1 | - | 1 | - |
|  | No | 0.69 (0.30–1.58) | 0.384 | 0.71 (0.26–1.94) | 0.504 | 0.55 (0.09–3.48) | 0.526 |
| Musculoskeletal diseases | No (ref) | 1 | - | 1 | - | 1 | - |
|  | Yes | 1.03 (0.65–1.61) | 0.916 | 0.59 (0.32–1.07) | 0.081 | 2.61 (1.12–6.10) | **0.027** |
| Self-assessment of current health status | Unsatisfied (ref) | 1 | - | 1 | - | 1 | - |
|  | Satisfied | 0.18 (0.09–0.38) | **<0.001** | 0.22 (0.09–0.58) | **0.002** | 0.14 (0.03–0.57) | **0.007** |
| Family conflicts | Yes (ref) | 0.27 (0.13–0.57) | **0.001** | 0.15 (0.05–0.40) | **<0.001** | 0.45 (0.12–1.67) | 0.231 |
|  | No | 1 | – | 1 | - | 1 | - |
| Social support | Low | 0.38 (0.17–0.85) | **0.019** | 0.12 (0.02–0.99) | **0.049** | 0.34 (0.12–0.97) | **0.041** |
|  | High (ref) | 1 |  | 1 | - | 1 | - |
